# *Cannabis sativa* Root Extract Exerts Anti-Nociceptive and Anti-Inflammatory Effects via Endocannabinoid Pathway Modulation In Vivo and In Vitro

**DOI:** 10.3390/ijms26188863

**Published:** 2025-09-11

**Authors:** Seo-Yul Jang, Hye-Lin Jin, Ga-Ram Yu, Dong-Woo Lim, Won-Hwan Park

**Affiliations:** 1 Department of diagnostics, College of Korean Medicine, Dongguk University, Dongguk-ro 32, Goyang-si 10326, Republic of Korea; jjanggu8316@gmail.com (S.-Y.J.); biojmail@naver.com (H.-L.J.); kalama2@dongguk.edu (G.-R.Y.); 2 Institute of Korean Medicine, Dongguk University, Dongguk-ro 32, Goyang-si 10326, Republic of Korea

**Keywords:** *cannabis* root, anti-nociception, analgesic effect, anti-inflammation, endocannabinoid pathway, CB1/CB2 ratio

## Abstract

*Cannabis sativa* root has traditionally been used to relieve pain and inflammation, but its pharmacological properties remain underexplored due to low levels of psychoactive cannabinoids. This study aimed to investigate the anti-inflammatory and antinociceptive effects of the ethyl acetate fraction of *Cannabis sativa* root (CSREA) using in vivo rodent pain models. Mice were subjected to formalin and acetic acid-induced nociceptive tests, while rats were evaluated using a carrageenan-induced paw edema model. CSREA significantly reduced pain-related behaviors in both early (0–10 min) and late phases (15–30 min) of the formalin test and decreased writhing responses in the acetic acid model. Notably, CSREA also improved survival rates following acetic acid injection. Inflammatory markers, including IL-6 and IL-1β, were significantly lowered in serum. Furthermore, CSREA suppressed paw edema and redness in the carrageenan-induced rat model, demonstrating dose-dependent anti-inflammatory efficacy comparable to diclofenac. CSREA also downregulated pain-related gene expression (*SCN9A*, *ASIC1A*, *TACR1*) and regulated key enzymes involved in endocannabinoid metabolism (*FAAH*, *MAGL*, *DAGL*), suggesting its role in the molecular modulation of pain pathways. These effects are likely mediated via modulation of the endocannabinoid system, particularly by rebalancing the CB1R/CB2R ratio. The findings suggest that CSREA holds promise as a natural therapeutic agent for managing pain and inflammation and warrants further investigation into its molecular mechanisms and long-term effects.

## 1. Introduction

Pain is a fundamental and complex sensory experience that plays a critical role in survival and well-being. It serves as a warning system that alerts individuals to potential or actual tissue damage, prompting protective responses [1]. Pain can be broadly categorized into acute and chronic pain [2]. Acute pain is typically a temporary response to a specific injury or illness, whereas chronic pain associated with various conditions such as neuropathic pain caused by damage to the nervous system, and inflammatory pain arises from the inflammatory response in damaged tissue [2]. The mechanisms underlying pain perception involve a complex interplay between the peripheral and central nervous system pathways. Nociceptors, specialized sensory neurons, detect harmful stimuli and transmit signals to the spinal cord and brain, where they are processed and interpreted as pain [3]. Effective management of pain arising from various causes—including musculoskeletal degenerative diseases, cancer, and neuropathic conditions—is essential for improving patients’ quality of life [4]. Given the multifactorial nature of chronic pain, a wide range of pharmacological agents—including opioids, NSAIDs, and adjuvant therapies—have been clinically used [4]. Opioids, which are used to treat moderate to severe pain, are known for their side effects, tolerance, and potential for dependency which limits long-term usage [4,5]. Therefore, efforts to develop new drugs to replace current agents are ongoing. In particular, many herbal medicine and herb-derived natural agents used for centuries are now being considered for drug development [6].

*Cannabis sativa* L., an annual plant of the family Cannabaceae, is considered one of the oldest cultivated crops and medicinal plant in human history [7]. The therapeutic potential of Cannabis was first scientifically validated in 1839 by William O’Shaughnessy, who demonstrated its analgesic anticonvulsant properties, leading to its use as a pharmaceutical agent [7].

Cannabis is a chemically complex plant, containing over 400 identified compounds, of which more than 60 are classified as cannabinoids [8]. Key cannabinoids include tetrahydrocannabinol (THC), cannabidiol (CBD), cannabichromene (CBC) and cannabinol (CBN) [9]. Among these, THC and CBD are the most prominent, with chemical structures analogous to endogenous cannabinoids present in the human body [10]. Cannabis extracts have been shown to have anti-inflammatory properties such as rheumatoid arthritis, cardiovascular disorders, and inflammatory bowel disease [11,12,13,14]. CBD has shown clinical efficacy in treating epilepsy, glaucoma, and depressive disorders, and has recently been suggested as a potential anti-inflammatory treatment for SARS-CoV-2 infection [15].

Cannabinoids interact with the endocannabinoid system primarily by modulating the cannabinoid receptor1 (CB1R, official gene symbol: *CNR1*), which plays a crucial role in the expression of peripheral nociceptor terminals, thereby contributing to the reduction in neuropathic pain and inflammation [10,16]. CB1 receptors, located primarily in the central nervous system, play a significant role in the modulation of pain perception [17,18]. The activation of CB1 receptors promotes the release of neurotransmitters involved in pain signaling, thus reducing nociceptive behaviors [18]. CB2 receptors (CB2R, official gene symbol: *CNR2*), primarily expressed on immune cells, are responsible for reducing inflammation [18,19]. Their activation leads to a decreased production of pro-inflammatory cytokines, which is critical in alleviating conditions such as carrageenan-induced paw edema [20] and may contribute to the reduction in inflammatory pain [21].

Cannabinoids control neuroplasticity by regulating the release and reuptake of neurotransmitters, and directly affect pain perception to exhibit anti-nociceptive effects, particularly in conditions such as arthritic pain and neuropathic pain [22]. In addition, cannabinoids exhibit neuroprotective effects under neuroinflammatory conditions, which might indirectly attenuate neuropathic pain [23].

Similarly, *Cannabis* roots (or root of *Cannabis sativa*, CR) have traditionally been used for treating inflammation, pain, fever, and joint pathologies [24]. CR extracts have been shown to inhibit inflammatory signaling pathways by suppressing cyclooxygenase-2 (COX-2) and inducible nitric oxide synthase (iNOS), resulting in the reduced production of pro-inflammatory cytokines [24]. Both in vitro and in vivo studies have demonstrated that CR extract can suppress the secretion of cytokines such as interleukin (IL)-6, tumor necrosis factor-alpha (TNF-α), and IL-1β in macrophages differentiated from THP-1 cells [24,25]. Another recent study has highlighted the potential of crude extracts of CR in pain management, particularly due to its antinociceptive effects, although the underlying mechanisms remain unexplored [26].

Our preliminary studies demonstrated that the ethyl acetate fraction of the 70% ethanol extract of CR showed superior anti-inflammatory activity compared to the hexane, butanol, and water fractions. Specifically, this fraction significantly reduced inflammation by modulating the JAK2–STAT3 pathway, which differs from the conventional NF-κB pathway commonly targeted by other natural compounds. Furthermore, we observed that this anti-inflammatory activity is associated with the inhibition of extracellular signal-regulated kinase (ERK) activation and downregulation of CB1 receptor expression. Notably, we also found that treatment with CR ethyl acetate extract restored endogenous cannabinoid 2-arachidonoylglycerol (2-AG) levels, suggesting its modulatory effects on the endocannabinoid system. Overall, through in vitro studies, we confirmed that CR exhibits anti-inflammatory properties mediated via the endocannabinoid system. However, research on the antinociceptive effects and mechanisms of CR fractions remains limited.

Therefore, the objective of this study is to further evaluate the anti-inflammatory and antinociceptive efficacy of ethyl acetate fraction of CR extract (CSREA) through animal experiments using mouse and rat pain models. Additionally, in vitro studies to demonstrate the mechanisms behind the antinociceptive effects of CSREA were conducted.

## 2. Results

### 2.1. Effect of CSREA on the Formalin Behavioral Test in Mouse Model

**Figure 1:**
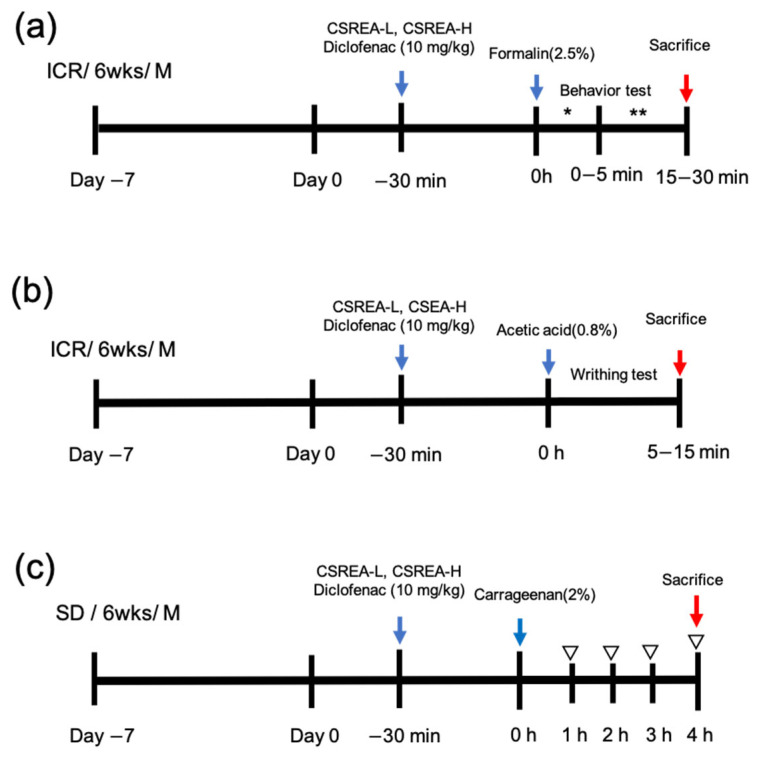
**Schematic representation of the in vivo pain models used in this study** (**a**) Formalin test in mice; (**b**) writhing test in mice; and (**c**) carrageenan-induced paw edema model in rats *: Phase I; **: Phase II; ▽: measurement of paw edema.

**Figure 2:**
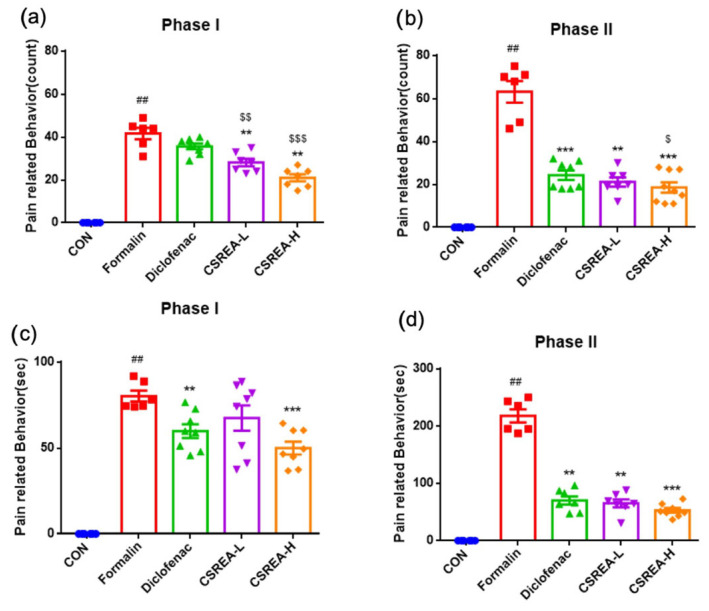
**The analgesic and anti-inflammatory effects of CSREA on the behavioral response of mice in the formalin test:** (**a**) phase I; (**b**) phase II pain behavior by scratch counts; (**c**) phase I and (**d**) phase II pain behavior by duration time. Data are expressed as mean ± SD ## *p* < 0.01 versus the CON group, *** *p* < 0.001, ** *p* < 0.01 versus the Formalin group and $$$ *p* < 0.001, $$ *p* < 0.01, $ *p* < 0.05 versus the Diclofenac group.

To confirm the anti-inflammatory effect of CSREA, we conducted a formalin test (Figure 1a). This test is a widely used animal model of pain characterized by biphasic pain response that is a tool for both acute and persistent pain mechanisms [27,28]. In the early phase (0–5 min after formalin injection), the number of scratch behaviors significantly increased in the formalin group compared to the control group (*p* < 0.001) (Figure 2a). In contrast to the formalin group, both the Diclofenac and CSREA-L (7.5 mg/kg) groups showed a decrease in the number of scratches, while the CSREA-H (15 mg/kg) group showed a significantly greater reduction (*p* < 0.05) (Figure 2a).

In the later phase (15–30 min after formalin injection), the number of scratches was significantly higher in the formalin group compared to the control group (*p* < 0.001) (Figure 2b). However, both the diclofenac group and CSREA-L group showed a decrease in the number of scratches compared to the formalin group (*p* < 0.001) (Figure 2b). Notably, the CSREA-H group exhibited a significantly greater reduction in the number of scratches than the formalin group (*p* < 0.001), and then the diclofenac group (*p* < 0.05) (Figure 2b). The duration of scratches followed a similar pattern to the number of scratches, showing a consistent reduction in CSREA-H (Figure 2c,d).

### 2.2. Anti-Inflammatory Effects of CSREA in Formalin-Induced Mouse Serum

**Figure 3:**
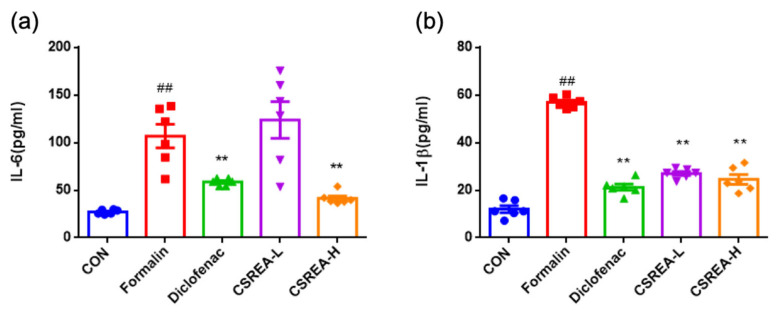
**Anti-inflammatory effect of CSREA assessed by serum analysis in mice after formalin test.** (**a**) IL-6 and (**b**) IL-1β levels in mouse serum. Data are expressed as mean ± SD ## *p* < 0.01 versus the CON group, ** *p* < 0.01 versus the Formalin group.

To investigate the ant-inflammatory effects of CSREA, we performed pro-inflammatory cytokine ELISA using serum samples (Figure 3). As a result, IL-6 levels in the formalin group were significantly elevated compared to the control group (*p* < 0.01) (Figure 3a). The Diclofenac and CSREA-H groups showed a significant reduction in IL-6 levels compared to the formalin group. But the CSREA-L groups did not exhibit a significant reduction as compared to the formalin group (Figure 3a). Notably, the CSREA-H group exhibited a significantly greater reduction in serum IL-6 levels compared to the CSREA-L group (*p* < 0.01) (Figure 3a). The serum IL-1β levels in the formalin group were significantly increased compared to the control group (*p* < 0.01) (Figure 3b). In contrast, the diclofenac, CSREA-L, and CSREA-H treatment groups all showed significant reductions in IL-1β levels compared to the formalin group (*p* < 0.01) (Figure 3b).

### 2.3. Effect of CSREA on the Acetic Acid-Induced Writhing Test in a Mouse Model

**Figure 4:**
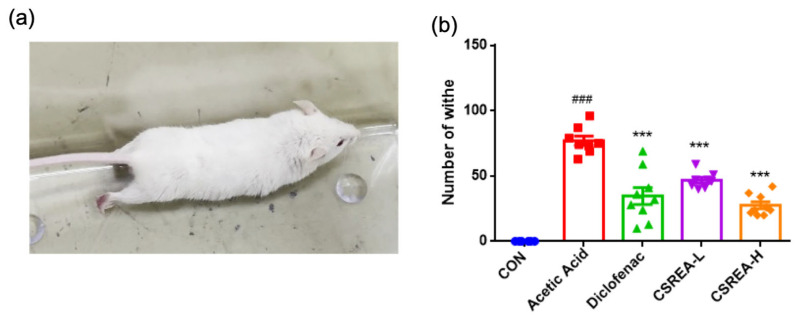
**Analgesic effect of CSREA assessed by writhing test in mice** (**a**) Mice showing writhing behavior after acetic acid injection. (**b**) Amount of writhing behavior in mice. Data are expressed as mean ± SD ### *p* < 0.001 versus the CON group, *** *p* < 0.001, versus the acetic acid group.

The writhing test using acetic acid as an inducer is widely used experimental model for assessing analgesic effect of drugs [29,30] (Figure 4a). We confirmed that the acetic acid group showed a significantly increased number of writhing responses compared to the control group (*p* < 0.001) (Figure 4b). On the contrary, all treatment groups exhibited a significantly decreased amount of writhing compared to the acetic acid group (*p* < 0.001) (Figure 4b).

### 2.4. Effect of CSREA on Survival in Mice Following Acetic Acid-Induced Model

After injection of acetic acid, the survival rate of mice in each group was evaluated (2 weeks after the test). The control groups showed a 100% survival rate, while the acetic acid group showed a 0% survival rate (Table 1). However, the CSREA-L and CSREA-H treatment groups showed 77.7% and 100% survival rates, respectively. The positive control group mice injected with diclofenac showed a survival rate of 44.4%.

### 2.5. Effects of CSREA on Carrageenan-Induced Inflammation in Rat Model

**Figure 5:**
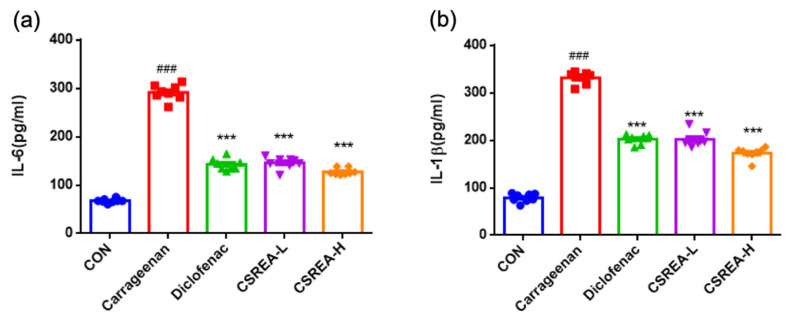
**Anti-inflammatory effect of CSREA by carrageenan in rat serum analysis** (**a**) IL-6 and (**b**) IL-1β levels in the rat serum. Data are expressed as mean ± SD. ### *p* < 0.001 versus the CON group, *** *p* < 0.001 versus the carrageenan group.

The carrageenan-induced in vivo model is widely used to induce hyperalgesia associated with acute inflammatory condition [31]. To evaluate the anti-inflammatory effects of CSREA, serum levels of IL-6 and IL-1β were quantified using ELISA. Serum IL-6 levels were significantly elevated in the carrageenan-induced group compared to the control group (*p* < 0.001) (Figure 5a). In contrast, the diclofenac-treated group exhibited a significant reduction in IL-6 levels relative to the carrageenan group (*p* < 0.001). Notably, both CSREA-L and CSREA-H treatment groups showed significant reduction in IL-6 levels as compared to the carrageenan-induced group (*p* < 0.001), with no apparent dose-dependent difference observed (Figure 5a). Similarly, serum IL-1β levels were significantly increased in the carrageenan group compared to the control group (*p* < 0.001) (Figure 5b). Conversely, the diclofenac-treated group showed significantly reduced IL-1β levels relative to the carrageenan group. Consistently, both CSREA-L and CSREA-H treatments led to significant reductions in IL-1β levels compared to the carrageenan group (Figure 5b).

### 2.6. Effects of CSREA on Carrageenan-Induced Hind Paw Edema in a Rat Model

**Figure 6:**
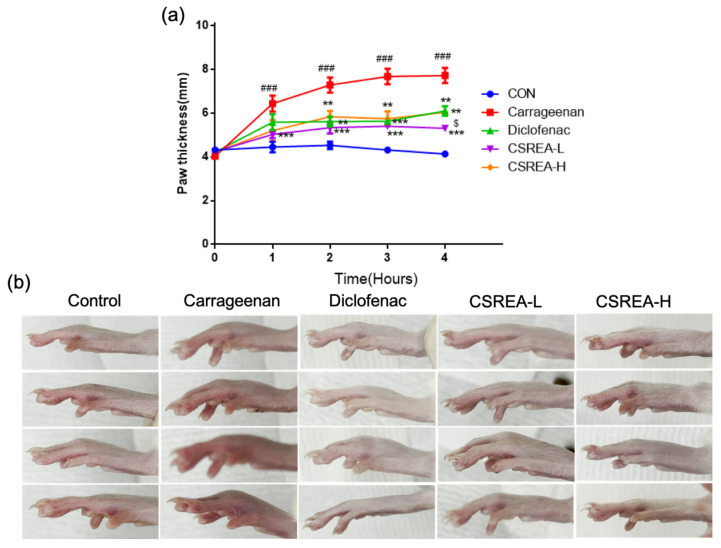
**Anti-inflammatory effect of CSREA on carrageenan-induced paw edema model** (**a**) Measurement of hind paw thickness. (**b**) Images of hind paw edema 4 h after carrageenan injection for comparing redness and thickness. Data are expressed as mean ± SD. ### *p* < 0.001 versus the CON group, *** *p* < 0.001, ** *p* < 0.01 versus the carrageenan group and $ *p* < 0.05 versus the diclofenac group.

We investigated the anti-inflammatory effect of the CSREA using the carrageenan-induced paw edema model. As a result of carrageenan injection, the paw thickness was significantly increased in the carrageenan group compared to the control group (*p* < 0.001) (Figure 6a).

In contrast, in the CSREA-L, CSREA-H and Diclofenac group, paw swelling was significantly suppressed (*p* < 0.001, *p* < 0.01 and *p* < 0.01, respectively). Particularly the CSREA-L significantly suppressed carrageenan induced edema compared to the other treatment groups (*p* < 0.001). Four hours after carrageenan injection, the carrageenan group showed visibly red and swollen paws than the control and other treatment groups (Figure 6b).

### 2.7. Anti-Inflammatory Effects of CSREA in a Carrageenan-Induced Inflammation Model in Rats

Histological analysis of hind paw section slide confirmed the presence of inflammation response at the site of carrageenan injection 4 h after administration. Specifically, we observed substantial differences in dermal edema among the experimental groups.

**Figure 7:**
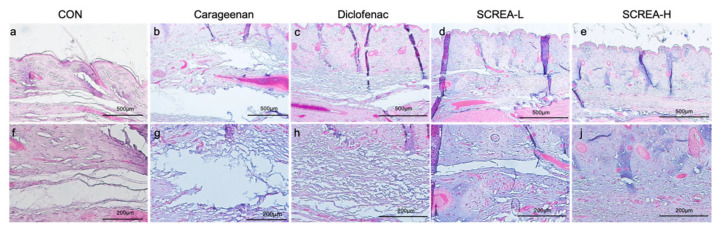
**Histopathological evaluation of paw dorsal tissue following carrageenan-induced inflammation.** Representative hematoxylin and eosin (H&E)-stained sections of paw skin are shown for each group: (**a**,**f**) control (CON), (**b**,**g**) carrageenan, (**c**,**h**) diclofenac-treated, (**d**,**i**) CSREA-L (low dose), and (**e**,**j**) CSREA-H (high dose). The upper panels (**a**–**e**) represent images taken at 40× magnification, while the lower panels (**f**–**j**) correspond to 100× magnification.

In the carrageenan group, marked expansion of the interstitial space was observed, indicative of pronounced dermal edema compared to the control group (Figure 7b,g). In the diclofenac group, the dermal structure appeared loosened, accompanied by a noticeable reduction in edema relative to the carrageenan group (Figure 7c,h).

In contrast, both the CSREA-L and CSREA-H groups exhibited only mild interstitial edema, which was significantly less pronounced than that in the carrageenan group (Figure 7d,e,i,j). Notably, in the CSREA-H group, no apparent dermal edema was observed, suggesting complete suppression of carrageenan-induced swelling (Figure 7e,j).

### 2.8. CSREA Regulates the Expression of Endocannabinoid-Related Genes in RA-Differentiated SH-SY5Y Cells

**Figure 8:**
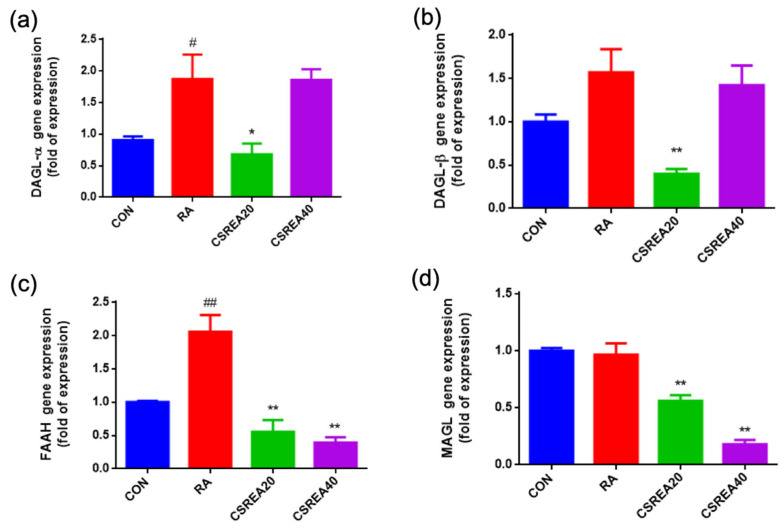
Effects of CSREA on the expression of endocannabinoid-related genes in RA-differentiated SH-SY5Y cells. RA: retinoic acid. Relative mRNA expression levels of (**a**) *DAGL-α*, (**b**) *DAGL-β*, (**c**) *FAAH*, and (**d**) *MAGL* were analyzed by RT-qPCR after 5 days of retinoic acid (RA)-induced differentiation followed by treatment with CSREA (20 or 40 μg/mL) for 6 h. Expression values were normalized to *PPIA* gene and are presented as fold change relative to the control group (CON). Data are shown as mean ± SEM. ## *p* < 0.01, # *p* < 0.05 versus the CON group, ** *p* < 0.01, * *p* < 0.05 versus the RA group.

To investigate the regulatory effects of CSREA on enzymes involved in endocannabinoid biosynthesis and degradation, the mRNA expression levels of *DAGL-α*, *DAGL-β*, fatty acid amide hydrolase (*FAAH*), and monoacylglycerol lipase (*MAGL)* were analyzed in retinoic acid (RA)-differentiated SH-SY5Y cells. RA treatment significantly increased *DAGL-α* expression compared to the control group (*p* < 0.05), indicating enhanced 2-AG biosynthetic capacity (Figure 8a). CSREA at 20 μg/mL significantly reduced *DAGL-α* expression relative to the RA group (*p* < 0.05), whereas the 40 μg/mL CSREA group showed a significant increase compared to the control group (*p* < 0.05). A similar trend was observed for *DAGL-β* expression (Figure 8b). RA treatment elevated *DAGL-β* levels compared to control group, and treatment with the CSREA at 40 μg/mL also increased expression level relative to the control group. Regarding the catabolic enzymes, RA markedly upregulated *FAAH* expression compared to the control group (*p* < 0.01; Figure 8c). Both CSREA treatments significantly reduced *FAAH* expression compared to the RA group (*p* < 0.01). Likewise, *MAGL* expression was significantly decreased in both CSREA-treated groups compared to the RA group (*p* < 0.01; Figure 8d).

These findings suggest that CSREA may modulate key signaling molecules of the endocannabinoid system by downregulating degradative enzyme and maintaining or enhancing biosynthetic enzyme expression, thereby contributing to increased endocannabinoid system activity.

### 2.9. CSREA Decreased Expression Level of Pain-Related Genes in SH-SY5Y Cells

**Figure 9:**
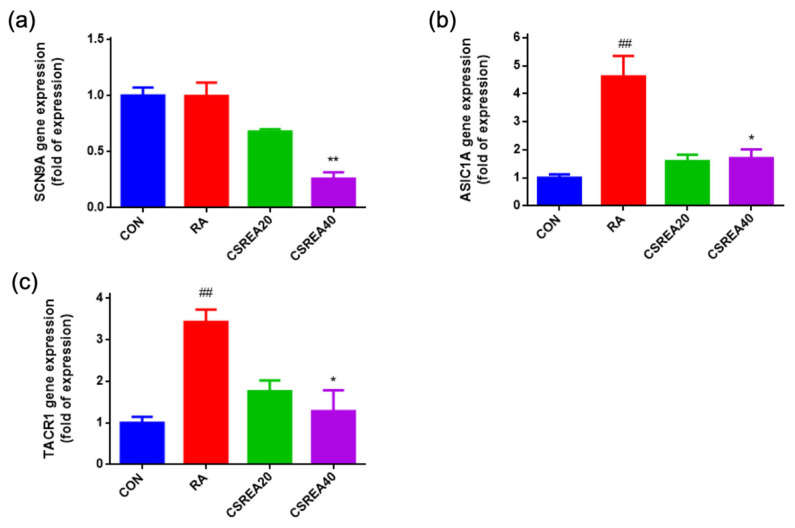
**Effect of CSREA on the expression of pain-related genes in RA-differentiated SH-SY5Y cells.** RA: retinoic acid. Relative mRNA expression levels of (**a**) *SCN9A*, (**b**) *ASIC1A*, and (**c**) *TACR1* were determined by RT-qPCR following treatment with 20 or 40 μg/mL CSREA for 6 h after 5-day retinoic acid (RA)-induced differentiation. Gene expression levels were normalized to *PPIA* and are presented as fold changes relative to the control (CON) group. Data are expressed as mean ± SEM. ## *p* < 0.01 versus the CON group, ** *p* < 0.01, * *p* < 0.05 versus the RA group.

To evaluate the effects of CSREA on pain-associated gene expression, mRNA levels of sodium voltage-gated channel alpha subunit 9 (*SCN9A*), acid-sensing ion channel 1 alpha (*ASIC1A*), and tachykinin receptor 1 (*TACR1*) were measured in RA-differentiated SH-SY5Y cells. The expression of *SCN9A* was slightly elevated in the RA-treated group compared to the control group. However, treatment with CSREA at 40 μg/mL significantly reduced *SCN9A* expression relative to the control group (*p* < 0.01; Figure 9a). *ASIC1A* expression was significantly increased following RA treatment compared to the control group (*p* < 0.01; Figure 9b). Co-treatment with CSREA at both 20 μg/mL and 40 μg/mL attenuated this upregulation, with the CSREA 40 μg/mL group showing a significant reduction compared to the RA group (*p* < 0.05). Similarly, *TACR1* expression was markedly elevated by RA treatment (*p* < 0.01 vs. control; Figure 9c). CSREA at 40 μg/mL significantly suppressed *TACR1* expression compared to the RA group (*p* < 0.05), while the CSREA 20 μg/mL group showed a slight, non-significant decrease. These results indicate that CSREA effectively downregulates the RA-induced expression of key nociceptive markers, including *SCN9A*, *ASIC1A*, and *TACR1*, in a dose-dependent manner, suggesting its potential modulatory role in pain-related gene regulation.

### 2.10. CSREA Regulates Endocannabinoid Receptors in SH-SY5Y Cells

**Figure 10:**
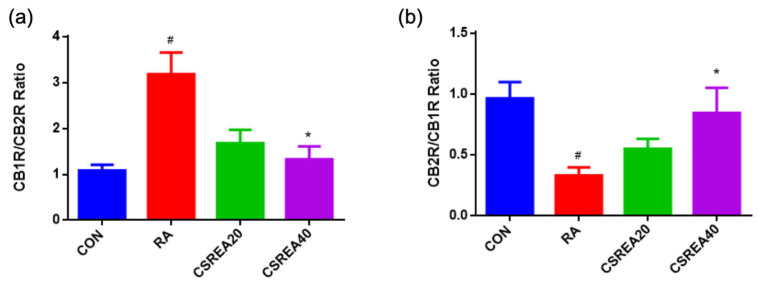
CB1R/CB2R (**a**) and CB2R/CB1R (**b**) ratios in RA-differentiated SH-SY5Y cells. RA significantly altered receptor balance compared to control, while CSREA treatment tended to restore the ratio in a dose-dependent manner. Data are mean ± SEM. # *p* < 0.05 vs. CON; * *p* < 0.05 vs. RA. RA: retinoic acid.

The relative balance between CB1R and CB2R is closely associated with cannabinoid-mediated modulation of neuronal excitability and neuroinflammation. The CB1R/CB2R ratio was significantly reduced in the RA-treated group compared to the control group (*p* < 0.05, Figure 10a). Treatment with CSREA at 20 μg/mL and 40 μg/mL decreased the CB1R/CB2R ratio, with the CSREA 40 μg/mL group showing a significant decrease compared to the RA group (*p* < 0.05). Conversely, the CB2R/CB1R ratio was markedly reduced in the RA group relative to the control group (*p* < 0.05, Figure 10b), reflecting an imbalance toward CB2R-dominant signaling. The CSREA treatment increased this ratio in a dose-dependent manner, and the CSREA 40 μg/mL group demonstrated a significant increase compared with the RA group (*p* < 0.05). These findings suggest that the increased CB1R/CB2R ratio due to RA-induced differentiation may indicate a shift toward a pro-inflammatory state and neuronal sensitization, whereas restoration of the ratio by CSREA may suggest rebalancing of neuroprotective and anti-inflammatory signaling.

## 3. Discussion

CBD, THC and the combination of both cannabinoids have been reported to exert a wide range of analgesic effects including nociceptive pain and neuropathic pain with distinct mechanism, which are potentially applicable for the treatment of severe chronic pain [32,33]. Despite the promising therapeutic applications, cannabis products and cannabinoids remain a controlled substance in many countries due to its psychoactive and addictive properties [34]. Meanwhile, CR, which contains only trace amounts of THC and CBD but is rich in bioactive compounds such as friedelin and epifriedelin [35], have been reported to exert multifaceted effects through various active ingredients [36]. Demonstrating the analgesic effect and mechanism of CR extract, in relation to the predicted bioactive compound, is necessary for its potential industrial or pharmaceutical application as a regulation-free alternative to conventional cannabis products.

In the present study, CSREA reduced Phase I pain-related behavior, which predominantly reflects direct peripheral nociceptor activation in the formalin test [37]. This early effect is compatible with a peripheral analgesic mechanism. Our neuronal cell data showed modulation of *SCN9A* (Nav1.7), *ASIC1A*, and *TACR1*, indicating reduced excitability and neuropeptide signaling (Figure 9). Concomitant alterations in *FAAH*/*MAGL* (Figure 8) and the CB1/CB2 receptor balance (Figure 10) further support enhanced endocannabinoid tone. Elevated AEA/2-AG would be expected to rapidly suppress nociceptor activity through presynaptic CB1-mediated inhibition of transmitter release and TRPV1 desensitization [38,39]. Taken together, these findings suggest that the attenuation of Phase I nocifensive behavior by CSREA may be mediated through modulation of the endocannabinoid system, leading to suppression of peripheral nociceptor excitability [40,41].

CSREA exhibited significant anti-inflammatory effects across various in vivo models. In the formalin-induced inflammation model, systemic IL-6 and IL-1β levels were markedly elevated in the formalin group but were significantly reduced by CSREA treatment, particularly at the higher dose (Figure 3). These results are consistent with behavioral outcomes observed during the late phase of the formalin test, where CSREA attenuated inflammatory pain (Figure 2). Similarly, in the carrageenan-induced inflammation model in rats, CSREA significantly reduced serum pro-inflammatory cytokines and paw edema, supporting its systemic anti-inflammatory efficacy (Figure 5 and Figure 6).

Histopathological analysis of H&E-stained tissue sections revealed severe dermal edema in the carrageenan-treated group, which was significantly reduced in the CSREA-treated group (Figure 7). Notably, the high-dose CSREA group exhibited only minimal interstitial swelling, indicating near-complete suppression of local inflammation. These findings suggest that CSREA provides local protection against tissue damage caused by the carrageenan-induced inflammatory response, as evidenced by the reduction in serum inflammatory markers.

Pro-inflammatory mediators produced by activated immune cells in damaged tissue or following nerve injury increase the resting membrane potential of neurons, lowering the threshold for activation and leading to nociceptor sensitization [42]. Therefore, the anti-inflammatory properties of CSREA demonstrated in our study may contribute to its pain-relieving effects. Inflammation and pain are closely linked through overlapping pathophysiological processes.

Pain is triggered by the recognition of harmful stimuli via nociceptors, which are specialized sensory neurons located as free nerve endings on Aδ and C fibers. These fibers possess distinct properties and transmit noxious signals to the cerebral cortex, where they are perceived as pain [43]. Although inflammatory and neuropathic pain have distinct etiologies, they share key mechanisms including altered ion channel expression, glial activation, and spinal sensitization. Understanding these overlapping pathways may help explain the transition from acute to chronic pain states [44].

Cannabinoids have been shown to inhibit C fiber-induced responses in dorsal horn neurons under normal conditions as well as in models of inflammation and nerve injury [45]. This effect is consistent with evidence demonstrating that cannabinoids reduce the expression of Fos protein-a marker of prolonged neuronal activation through CB1 and CB2 receptor-mediated pathways in various models of chronic pain [46,47,48]. However, as we noted, CSREA contains trace amounts of cannabinoids, which may not sufficiently account for explaining the analgesic properties of CSREA.

In our previous study, we hypothesized that the endocannabinoid system might be modulated by bioactive compounds derived from CR, which are not classified as cannabinoids (data not published). Unique phytochemicals of CR such as friedelin, epifriedelinol, and stigmasterol, which are known for their potential anti-inflammatory and analgesic effects [35,49,50], and may influence downstream signaling pathways. In addition, these non-cannabinoid compounds may affect the cannabinoid system directly (by interacting with traditional cannabinoid receptors (CB1 and CB2) or non-classical cannabinoid receptor- transient receptor potential cation channel subfamily V member 1 (TRPV1) and G protein-coupled receptor 55 (GPR55) [51] or indirectly (by modulating the endocannabinoid synthesis/degradation to control endocannabinoid levels, or even may affect other unknown pathways) to exhibit analgesic efficacy [45]. For instance, β-caryophyllene, a non-cannabinoid compound, is known to act as a CB2 receptor agonist, directly interacting with CB2R [52]. Moreover, non-cannabinoid agents, including certain non-steroidal anti-inflammatory drugs (NSAIDs), have been reported to exert indirect analgesic effects by inhibiting the metabolic degradation of endocannabinoids, thereby enhancing local endocannabinoid tone [53].

In our previous study, we demonstrated that CSREA regulates the balance of CB1 and CB2 receptors and reduces inflammatory cytokine levels at the in vitro level (Not published). The delicate changes in expression pattern of CB1 and CB2 receptors have been observed in various conditions, and are considered crucial in the pain regulation [54]. To control the balance of the receptor activity, various agonists and antagonists targeting cannabinoid receptors have been tested across different types of pain and pathological conditions [55]. Moreover, CB2-linked anti-inflammatory effects may contribute more prominently to Phase II.

2-AG, a key endocannabinoid, plays a significant role in modulating inflammation and nociception via CB1 and CB2 receptors [56,57]. 2-AG is synthesized on demand and rapidly degraded by hydrolytic enzymes, and its levels can be influenced by signaling lipids in central nervous systems which are critical regulators of neurotransmitter release. This biological process is involved in both physiological modulation of pain and pathological condition of neuroinflammation [39,58,59]. In this context, the enzymatic degradation of 2-AG by enzymes such as FAAH and MAGL is equally important in regulating its levels and, consequently, its effects on pain and inflammation [60,61]. Therefore, inhibition of these enzymes may increase 2-AG concentrations, enhancing anti-nociceptive and anti-inflammatory responses [60,62,63].

CSREA significantly reduced lipopolysaccharide (LPS)-induced elevations in 2-AG levels, accompanied by a marked decrease in pro-inflammatory cytokine expression. Conversely, in the absence of LPS stimulation, CSREA increased 2-AG levels, indicating a potential role in modulating endocannabinoid synthesis or degradation. This homeostatic effect of CSREA on 2-AG level may contribute to both its analgesic and anti-inflammatory potential. As demonstrated by the carrageenan-induced in vivo model, CSREA has the potential to inhibit local inflammation and reduce hind paw edema and serum cytokine levels, which may contribute to the attenuation of inflammatory pain responses. Furthermore, the therapeutic potential of CSREA may not be limited to acute pain response; it may attenuate chronic pain caused by neuronal inflammation or injury-associated neuropathic pain.

In addition to the receptor-based mechanisms, we have conducted cell-based experiments focusing on inflammation-related cytokines and nociceptive effect of CSREA. Our results indicate that treatment with CSREA led to a reduction in the levels of key pro-inflammatory cytokines, including IL-6 and IL-1β (Figure 3 and Figure 5). This suggests that the anti-inflammatory effects of the extract may extend to modulating cellular responses, particularly in immune cells, and reducing inflammation at the molecular level.

CSREA effectively counteracted RA-induced receptor imbalance by restoring the CB1R/CB2R ratio (Figure 10a,b). The transcript expression pattern of CB1R and CB2R is highly tissue-specific, and the ratio reflects the differentiation or developmental stages in neurons or immune cells [64,65]. This rebalancing suggests that CSREA modulates endocannabinoid receptor dynamics, potentially enhancing CB1R-mediated neuroprotective signaling and regulating CB2R-mediated immune responses. Such modulation may underlie the antinociceptive and anti-inflammatory effects observed in this study.

However, this study has several limitations. First, it does not identify the specific endocannabinoid receptors through which CSREA may exert its effects. The hypothesis that CSREA exhibits anti-nociceptive effects by modulating endocannabinoid receptors requires validation using rigorous experimental models, including receptor-specific knockout in vivo studies. Furthermore, in vivo measurements of cannabinoid levels—both local and systemic—are needed to confirm the correlation between altered expression of degradation enzymes and the observed analgesic effects. While diclofenac was used as a reference drug, a direct comparison using equipotent doses was not performed in this study. Future investigations will aim to establish pharmacological equivalence between CSREA and diclofenac by incorporating additional dose-ranging groups to better elucidate the relative efficacy and optimal dosing strategy of CSREA.

It is important to acknowledge several limitations in the present study that should be considered when interpreting its translational potential. Our experimental design employed a preventative analgesic model, wherein CSREA was administered prior to the induction of pain. While this approach is widely used for the initial screening of a compound’s intrinsic antinociceptive properties [66]; however, this design does not fully replicate the typical clinical scenario in humans, where analgesic treatments are administered after pain is already established. Future studies should therefore incorporate therapeutic models (i.e., administration of CSREA after the pain stimulus) to assess its efficacy in a more clinically relevant context.

This study presents evidence that CSREA possesses analgesic effects via modulating the endocannabinoid system in vivo. However, to fully elucidate the mechanisms underlying these effects, future studies should focus on investigating the specific signaling pathways involved in the antinociceptive effect of CSREA, their interaction with CB1 and CB2 receptors, and the modulation of 2-AG levels through the inhibition of *FAAH* and *MAGL*. Additionally, further in vitro and in vivo studies are needed to explore the long-term effects of CSREA on chronic pain and neuroinflammatory diseases. Additional positive controls, such as opioids, will be essential to better distinguish whether the antinociceptive effects of CSREA are mediated predominantly through neurogenic or inflammatory mechanisms. A comprehensive understanding of the pharmacological profile of the CSREA, including the role of *FAAH* and *MAGL* in the 2-AG pathway, will be essential in advancing its clinical applications.

## 4. Materials and Methods

### 4.1. Animal

For the evaluation of formalin and acetic acid-induced pain behavior, male ICR mouse, aged six weeks (body weight 25–30 g), were purchased from OrientBio, Korea Inc. (Seongnam, Republic of Korea). Animals were acclimated for a week under standard conditions with ad libitum access to food and water. For the assessment of carrageenan-induced paw edema and inflammation, six-weeks old male Sprague–Dawley rats (body weight 220 ± 20 g) were purchased from OrientBio (Seongnam, Republic of Korea). In all experiments, each mouse was subjected to a single experiment, and no animals were used in more than one protocol.

All mice and rats were housed in a 12 h light and dark cycle at a constant temperature of 22 ± 3 °C, humidity of 50 ± 5% with free access to water and a chow diet (Feedlab, Guri, Gyeonggi-do, Republic of Korea). This study was approved by the ‘Institutional Animal Care and Use Committee’ of the Dongguk University (IACUC-2024-050-01) and executed in strict accordance with the recommendations of the “Guide for the Care and Use of Laboratory Animals”.

Group allocations were known to the researcher throughout the study, including during allocation, experimentation, outcome assessment, and data analysis. Therefore, blinding was not implemented.

Due to the lack of available data on the expected effect size of CSREA in the formalin-induced pain model, it was not feasible to perform a priori power analysis. Therefore, no formal sample size calculation was conducted. Instead, the group size was determined based on previous studies using similar animal models and experimental endpoints, which demonstrated statistically significant outcomes with comparable group sizes. Accordingly, nine animals per group were selected for this study.

All animal handling was conducted by trained personnel, with procedures designed to minimize stress on the animals. Animals showing signs of illness, injury, or abnormal baseline behavior prior to the experiment were excluded from the study. No animals were excluded after treatment initiation.

### 4.2. Extraction of CSREA from Cannabis sativa L. Root

CR was provided from The Korean Cannabis Industry Association (Andong, Republic of Korea). The *C. Sativa* L. root separated from the plant was washed and dried. Two hundred grams of ground roots were extracted with 3 L of 70% ethanol for 3 days at room temperature. The crude extract of *C. Sativa* roots was concentrated using a rotary evaporator (Buchi, Flawil, Switzerland), and further sequential fractionated using hexane and ethyl acetate solvent, respectively, using an extraction funnel. Harvested fractionations were evaporated. The resulting fractions were evaporated and dried. CSREA was dissolved in DMSO and filtered using 20 μm syringe filter before use. Supplementary Table S1 summarizes the major known compounds from *Cannabis sativa* root, together with their reported concentrations and associated toxicity or safety information.

### 4.3. Formalin Test

A total of forty-five mice were randomly divided into 5 groups of 9 mice, respectively. Formalin injection was diluted with PBS, and a 2.5% (*v*/*v*) formalin solution was prepared. The mice were acclimated to the observation chamber for 30 min. After 30 min, 10 µL of 2.5% formalin solution was injected into the plantar surface of the right hind paw of the mice. Mice were pre-treated with PBS (Phosphate-Buffered Saline, i.p.), diclofenac (10 mg/kg, i.p), CSREA 7.5 mg/kg, i.p. (CSREA-L), CSREA 15 mg/kg, i.p. (CSREA-H), 30 min before injection of formalin. The dosages employed in the in vivo experiments were established based on preliminary testing for CSREA and on previously published studies for diclofenac [30,67]. The observation of pain-related behavior was divided into two phases. The first phase (Phase I) is the direct activity of neurogenic inflammation that directly stimulates nerve fibers and the second phase (Phase II), known as inflammatory pain results from the release of inflammatory mediators. After 5 min formalin injection, the mice were placed in an observation chamber and observed for 0–10 min (phase I) and 15–30 min. (phase II). The pain-related behaviors in terms of biting, licking, shaking, and lifting the formalin-injected paw were measured by inspecting recorded video.

### 4.4. Writhing Test

To evaluate the analgesic effect of CSREA, an acetic acid-induced writhing test was performed. Total forty-five mice were randomly divided into 5 groups, each consisting of 9 mice for the writhing test. To conduct this experiment, mice were intraperitoneally (i.p.) administered PBS, CSREA-L, CSREA-H or diclofenac (10 mg/kg, i.p.). Thirty minutes later, an additional injection of 0.8% acetic acid (10 mL/kg, i.p.) was administered. Five minutes after the injection, the amount of writhing was measured for 10 min.

### 4.5. Evaluation of Carrageenan-Induced Hind Paw Edema

Rat hind paw edema was induced by carrageenan using a previously described method [31]. Briefly, 20 µL volume of 2% carrageenan (Sigma, St. Louis, MO, USA) was administered via subcutaneous injection into the dorsum of the rat’s right hind paw. Total forty rats were randomly divided into five groups, each consisting of eight rats for the edema test. Thirty minutes prior to the injection of carrageenan, rats were pretreated with diclofenac (10 mg/kg, i.p.), CRSEA-L, CSREA-H and PBS (Phosphate-Buffered Saline, i.p.). The volume of intraplantar paw edema was measured using calipers at before and 0, 1, 2, 3 and 4 h after the injection.

### 4.6. Biochemical Analysis of Serum and Tissue Collection

At 4 h after the of 2% carrageenan injection, rats were anesthetized using isoflurane (Ifran, Hana Pharm Co., Hwaseong, Republic of Korea). The blood samples (~1 mL) were collected from abdominal aorta during the sacrifice and allowed to clot at room temperature for 1 h. After the clot formation, the samples were centrifuged at 3000 rpm for 15 min. The resulting serum was collected and stored in a deep freezer at −80 °C. The mouse serum level of IL-6 and IL-1β were determined by a Quantikine ELISA kit (R&D systems, Minneapolis, MN, USA) and the rat serum level was determined Rat IL-6 Elisa kit (Invitrogen, Waltham, MA, USA).

For histopathologic analysis, right dorsal hind paw of each rat was dissected after 4 h of 2% carrageenan injection. The samples underwent pre-treatment to prepare for histological analysis.

### 4.7. Cell Culture and Neuronal Differentiation

SH-SY5Y human neuroblastoma cells were routinely maintained in Dulbecco’s modified Eagle’s medium (DMEM; Gibco, Grand Island, NY, USA) supplemented with 10% heat-inactivated fetal bovine serum (FBS; hyclon, Logan, UT, USA) and 100 U/mL penicillin-streptomycin (Gibco). Cells were incubated at 37 °C in a humidified atmosphere containing 5% CO_2_. The culture medium was refreshed daily, and cells were subcultured once cells reached approximately 80% confluence.

For differentiation to neuronal phenotype, cells were seeded at a concentration 1 × 10^6^ cells/mL into 6 well plate.

After 24 h of initial attachment, the growth medium was replaced with differentiation medium consisting of DMEM containing 3% heat-inactivated FBS and 10 μM all-trans retinoic acid (Acros Organics, Thermo Fischer Scientific, Waltham, MA, USA). Cells were incubated for 5 days and the medium was changed every other day.

On the fifth day of differentiation, cells were subjected to the following treatments for 6 h: (1) untreated control (differentiation medium), (2) 10 μM RA alone, (3) 10 μM RA combination with 20 μg/mL CSREA, or (4) 10 μM RA combination with 40 μg/mL CSREA.

### 4.8. Gene Expression Analysis

The total RNAs were isolated from SH-SY5Y cell using TRIzol^®^ reagent (Invitrogen, Carlsbad, CA, USA) according to the manufacturer’s instructions. Complementary DNA (cDNA) was synthesized from 1 μg of total RNA using AccuPower^®^ RT PreMix (Bioneer, Daejeon, Republic of Korea) following the manufacturer’s instructions. Quantitative real-time Polymerase Chain Reaction (qPCR) was conducted using the LightCycler^®^ 96 Instrument (Roche Diagnostics, Mannheim, Germany) with the AccuPower^®^ 2X GreenStar™ qPCR Master Mix (Bioneer) in accordance with the supplier’s instruction. Gene expression levels were normalized to Peptidylprolyl Isomerase A (*PPIA*) expression. Primer sequences are listed in Table 2.

### 4.9. Histological Evaluation of Edema in Rat Paw Tissue

Paw tissues were collected 4 h after carrageenan injection and fixed in 4% neutral buffered formalin for 24 h at room temperature. To remove bone components, the fixed tissues were decalcified in 10% ethylenediaminetetraacetic acid (EDTA, pH 7.4) at room temperature for 24 h using a commercial decalcifying solution (Cat#: D-0818, Sigma, St. Louis, MO, USA). After complete decalcification, the tissues were dehydrated through a graded ethanol series, cleared in xylene, and embedded in paraffin.

Serial sections of 4 μm thickness were prepared using a microtome and mounted on glass slides. The sections were deparaffinized, rehydrated, and stained with hematoxylin for nuclear visualization and eosin for cytoplasmic contrast. Histological changes in tissue architecture were examined under a light microscope system with camera (DMI 6000, Leica, Wetzlar, Germany).

### 4.10. Statistical Analysis

Data are presented as the mean ± standard deviation (SD) or standard error of the mean (SEM). Statistical analyses were performed using GraphPad Prism 6 (GraphPad, San Diego, CA, USA). For multiple group comparisons, the nonparametric Kruskal–Wallis test followed by Dunn’s post hoc test was used. For pairwise comparisons, Student’s *t*-test was employed. A *p*-value < 0.05 was considered statistically significant.

## 5. Conclusions

This study provides evidence for the in vivo analgesic and anti-inflammatory effects and underlying mechanism of CSREA in vitro. Our results from the formalin and writhing tests demonstrate that CSREA significantly reduced nociceptive pain-related behaviors and inflammatory cytokine levels indicating strong anti-nociceptive properties in a dose-dependent manner. In addition, CSREA markedly reduced paw edema in the carrageenan-induced rat model, suggesting its potential as a natural product with anti-inflammatory activity. These effects are likely mediated through modulation of the endocannabinoid system, particularly by altering cannabinoid levels as demonstrated in the in vitro model.

## Figures and Tables

**Table 1:** Survival analysis following the acetic acid-induced mouse writhing test over 2 weeks.

Group	Number of Survived Subjects/Total Subjects	Survival Rate
Control	9/9	100%
Acetic acid	0/9	0%
Diclofenac	4/9	44.4%
CSREA-L	7/9	77.7%
CSREA-H	9/9	100%

**Table 2:** List of target genes and their corresponding primer sequences employed in RT-qPCR analysis (Homo sapiens).

**Gene**	**Acceesion No.**	**Primer Forward Sequence** **(5′-3′)**	**Primer Reverse Sequence** **(5′-3′)**
*PPIA*	NM_021130.5	GCCGAGGAAAACCGTGTACT	TGTCTGCAAACAGCTCAAAGGA
*FAAH*	NM_001441.3	TCAGCTTTCCTCAGCAACAT	CCGCAGACACAACTCTTCTT
*DAGLα*	NM_006133.3	CTTCCTCTTTCTCCTGCATACC	TGGCTTGACCCTCCTCTAA
*DAGLβ*	NM_139179.4	TCAGCATGAGAGGAACGATTT	GGCTCTTGGTTGTTCCTGATA
*MAGL*	NM_00138315.1	CTCATTTCGCCTCTGGTTCT	GAAGACGGAGTTGGTGACTT
*SCN9A*	NM_002977.4	GTCTCCCTGGTTGATGGACG	TGATTGGTCGTGCCCTCTGG
*TACR1*	NM_001058.4	ATGACAGGTTCCGTCTGGGC	TACACACTGCCCTGGGTCTG
*ASIC1α*	NM_001412756.1	AGCGGCTGTCTCTGAAGC	AGCTCCCCAGCATGATACAG
*C* *NR* *1*	NM_001370547.1	CGTCGTTCAAGGAGAATGAGG	TGCCGATGAAGTGGTAGGAAG
*C* *NR* *2*	NM_001841.3	CGTGGCTGTGCTCTATCTGA	AGCCAGCTCAGCAGGTAGTC

## Data Availability

All data are contained in this article.

## Supplementary Materials

The following supporting information can be downloaded at: https://www.mdpi.com/article/10.3390/ijms26188863/s1. References [68,69,70,71,72,73,74,75,76] are cited in Supplementary Materials.

## Abbreviations

The following abbreviations are used in this manuscript:

CSREA	*Cannabis sativa* root ethyl acetate fraction
CR	*Cannabis* root
CBD	Cannabidiol
THC	Tetrahydrocannabinol
CBC	Cannabichromene
CB1R	Cannabinoid receptor1
CB2R	Cannabinoid receptor2
iNOS	Inducible nitric oxide synthase
COX-2	Cyclooxygenase-2
TNF-α	Tumor necrosis factor-alpha
IL-6	Interleukin-6
2-AG	2-arachidonoylglycerol
MAGL	Monoacylglycerol lipase
FAAH	Fatty acid amide hydrolase
DAGL-α	Diacylglycerol lipase alpha
DAGL-β	Diacylglycerol lipase beta
ASIC1A	Acid-sensing ion channel 1a
SCN9A	Sodium voltage-gated channel alpha subunit 9
TACR1	Tachykinin receptor1
PPIA	Peptidylprolyl isomerase A
