# Supplementary material for: Cannabis sativa Root Extract Exerts Anti-Nociceptive and Anti-Inflammatory Effects via Endocannabinoid Pathway Modulation In Vivo and In Vitro
Source: Int J Mol Sci. 2025 Sep 11;26(18):8863. doi: 10.3390/ijms26188863 (PMC12470016; doi:10.3390/ijms26188863)
Supplement: Supplementary file 1 [file ijms-26-08863-s001.zip › ijms-3821048-supplementary.pdf]

Supplementary Table S1. List of compounds, reported contents, and their reported toxicity/safety issue contained in Cannabis roots

Major compounds in Cannabis root	Concentration	Toxicity/Safety issue	Reported biological activity
Friedelin	7.5-12.8mg/kg	N/R	Anti-inflammatory, Analgesic, antipyrectic, anti-viral, anti-oxidant [1]
Epifriedelanol	14.5-21.3mg/kg	N/R	Anti-inflammatory, anti-oxidant [2]
Carvone*	893.36 mg/kg	Low toxicity with rare cases of contact allergy; in animal studies, doses ≥30 mg/kg/day caused kidney changes in males, but no carcinogenicity or genotoxicity was observed.	Anti-microbial, anti-oxidant, anti-inflammatory [3]
Dihydrocarvone*	267.95 mg/kg	Low acute toxicity, with LD50 values above 5 g/kg in rats and rabbits; high-dose (subcutaneous) administration in mice produced anticonvulsant and sleep-related effects.	Antimicrobial [4]
Cannabisativine	2.5mg/kg	N/R	N.R
Anhydrocannabisativine	0.3mg/kg	N/R	N.R
β-Sitosterol	0.687mg/kg	No genotoxicity was seen in mice, but long-term intraperitoneal injections in rats caused mild liver and kidney lesions.	Anti-inflammatory, Anti-microbial, anti-oxidant, analgesic [5]
Campesterol	0.172mg/kg	In a study on rabbits was not irritating. Oil-derived mixture caused very slight and reversible erythema.	Anti-nociceptive, anti-inflammatory, anti-oxidant [6,7]
Stigmasterol	0.202mg/kg	In a two-generation study, no reproductive or developmental toxicity was observed in rats at high doses.	Anti-inflammation, analgesic, immunomodulation [8]
N-(p-hydroxy-b-phenylethyl)-p-hydroxy-trans cinnamamide	1.6 mg/kg	N/R	Anti-inflammation [9]

* Higher yields of carvone and dihydrocarvone due to hexane-based extraction and large-scale processing.

1. Radi, M.H.; El-Shiekh, R.A.; El-Halawany, A.M.; Abdel-Sattar, E. Friedelin and 3β-friedelinol: pharmacological activities. *Revista Brasileira de Farmacognosia* **2023**, *33*, 886-900.
2. Kobtrakul, K.; Rani, D.; Binalee, A.; Udomlarp, P.; Srichai, T.; De-Eknamkul, W.; Vimolmangkang, S. Elicitation enhances the production of friedelin and epifriedelanol in hairy root cultures of Cannabis sativa L. *Frontiers in Plant Science* **2023**, *14*, 1242584.
3. Pina, L.T.; Serafini, M.R.; Oliveira, M.A.; Sampaio, L.A.; Guimaraes, J.O.; Guimaraes, A.G. Carvone and its pharmacological activities: A systematic review. *Phytochemistry* **2022**, *196*, 113080.
4. Porto, C.; Stüker, C.Z.; Mallmann, A.S.; Simionatto, E.; Flach, A.; Canto-Dorow, T.d.; Silva, U.F.d.; Dalcol, I.I.; Morel, A.F. (R)-(-)-carvone and (1R, 4R)-trans-(+)-dihydrocarvone from poiretia latifolia vogel. *Journal of the Brazilian Chemical Society* **2010**, *21*, 782-786.
5. Saeidnia, S.; Manayi, A.; Gohari, A.R.; Abdollahi, M. The story of beta-sitosterol-a review. **2014**.
6. Nazir, S.; Ahmad, I.; Mobashar, A.; Sharif, A.; Shabbir, A.; Chaudhary, W.A. Mechanistic evaluation of antiarthritic and anti-inflammatory effect of campesterol ester derivatives in complete Freund’s adjuvant-induced arthritic rats. *Front. Pharmacol.* **2024**, *14*, 1346054.
7. de Morais Oliveira-Tintino, C.D.; da Silva, F.E.; Santiago, G.M.; das CL Pinto, F.; Pessoa, O.D.; da Fonseca, A.M.; Paulo, C.L.; Dos Santos, H.S.; Marinho, M.M.; Dos Santos, J.L. Molecular docking and antibacterial activity of campesterol derivatives against Staphylococcus aureus, Escherichia coli and Pseudomonas aeruginosa multiresistant strains. *Chemistry & Biodiversity* **2025**, *22*, e202401073.
8. Bakrim, S.; Benkhaira, N.; Bourais, I.; Benali, T.; Lee, L.-H.; El Omari, N.; Sheikh, R.A.; Goh, K.W.; Ming, L.C.; Bouyahya, A. Health benefits and pharmacological properties of stigmasterol. *Antioxidants* **2022**, *11*, 1912.
9. Ryz, N.R.; Remillard, D.J.; Russo, E.B. Cannabis roots: a traditional therapy with future potential for treating inflammation and pain. *Cannabis and cannabinoid research* **2017**, *2*, 210-216.
